# Human tumor necrosis factor (TNF)-alpha-induced protein 8-like 2 suppresses hepatocellular carcinoma metastasis through inhibiting Rac1

**DOI:** 10.1186/1476-4598-12-149

**Published:** 2013-11-26

**Authors:** Xuelei Cao, Li Zhang, Yongyu Shi, Yue Sun, Shen Dai, Chun Guo, Faliang Zhu, Qun wang, Jianing Wang, Xiaoyan Wang, Youhai H Chen, Lining Zhang

**Affiliations:** 1 Department of Immunology, Shandong University School of Medicine, 44# Wenhua Xi Road, Jinan 250012, China; 2 Department of Clinical Laboratory, Shandong Provincial Qianfoshan Hospital, Jinan China; 3 Department of Pathology and Laboratory Medicine, University of Pennsylvania School of Medicine, Philadelphia, USA

**Keywords:** TIPE2, HCC, Invasion, Metastasis, Rac1

## Abstract

**Background:**

Tumor invasion and metastasis are the major reasons for leading death of patients with hepatocellular carcinoma (HCC). Therefore, to identify molecules that can suppress invasion and metastasis of tumor will provide novel targets for HCC therapies. Tumor necrosis factor (TNF)-alpha-induced protein 8-like 2, TIPE2, is a novel immune negative molecule and an inhibitor of the oncogenic Ras in mice but its function in human is unclear. Our previous research has shown that TIPE2 is downregulated in human primary HCC compared with the paired adjacent non-tumor tissues.

**Results:**

In present study, we provide evidence that TIPE2 inhibits effectively human hepatocellular carcinoma metastasis. The forced expression of TIPE2 in HCC-derived cell lines markedly inhibits tumor cell growth, migration and invasion *in vitro* and suppresses growth and metastasis of HCC *in vivo*. Clinical information from a cohort of 112 patients reveals that loss or reduced expression of TIPE2 in primary HCC tissues is significantly associated with tumor metastasis. Mechanically, TIPE2 inhibits the migration and invasion through targeting Rac1 and then reduces F-actin polymerization and expression of matrix metallopeptidase 9 (MMP9) and urokinase plasminogen activator (uPA).

**Conclusion:**

Our results indicate that human TIPE2 is endogenous inhibitor of Rac1 in HCC by which it attenuates invasion and metastasis of HCC. The data suggest that TIPE2 will be a new target for HCC therapy.

## Background

Primary hepatocellular carcinoma (HCC) is one of the most common cancers and the third leading cause of death from cancer worldwide [1]. High vascular invasion and metastasis potential, and recurrence even after surgical resection contribute to the poor prognosis of patients with HCC [2]. Therefore, to identify molecules that can suppress invasion and metastasis will provide novel targets for HCC therapies.

TIPE2, tumor necrosis factor-alpha-induced protein 8 (TNFAIP8)-like 2 (TNFAIP8L2), is a newly described immune negative regulator and belongs to the TNFAIP8 family [3]. It maintains immune homeostasis via regulating negatively both the innate and adaptive immunity in mice [3]. Its deficiency in mice leads to multi-organ inflammation [3] and increases the cerebral volume of infarction and neurological dysfunction in experimental stroke [4]. TIPE2 is downregulated in patients with chronic inflammatory diseases such as systemic lupus erythematosus and hepatitis, and its expression inversely correlates with disease progression [5,6]. The high-resolution crystal structure reveals that TIPE2 contains a large, hydrophobic central cavity, which appears to be a mirror image of the death effector domain (DED) [7]. Murine TIPE2 is associated with caspase-8 and inhibits activating protein (AP)-1 and nuclear factor (NF)-κB activation while it promotes Fas-induced apoptosis [3]. MurineTIPE2 also binds directly and block Rac1GTPases to dictate the strengths of phagocytosis and oxidative burst in innate immune [8] and to controls innate immunity to RNA in mice [9]. In addition, TIPE2 binds to Ras-interacting domain of the RalGDS family of proteins to prevent Ras from forming an active complex. Consequently, TIPE2 overexpression induces cell death and significantly inhibits Ras-induced tumorigenesis in mice [10]. The results from mice suggest that TIPE2 is not only involved in inflammation but also in cancer.

Although murine TIPE2 has been partly characterized, human TIPE2 is largely unknown. Unlike murine TIPE2 that preferentially expresses in hematopoietic cells [3,11], human TIPE2 also does in a wide variety of non-hematopoietic cell types, including hepatocytes [12]. Recently, our previous research has shown that TIPE2 expression is completely lost or significantly downregulated in human primary HCC but is high in all the paired adjacent non-tumor tissues [10]. However, the role and underlining mechanisms of human TIPE2 in development and progression of HCC remain to be investigated.

In present study, we analyze the relation of loss or reduced expression of TIPE2 in primary HCC tissues with clinicopathological characteristics, investigate the role of TIPE2 in tumor growth, migration and invasion of HCC *in vitro* and *in vivo* and further explore its underlining mechanisms. Our results indicate that human TIPE2 is endogenous inhibitor of Rac1 in liver by which it attenuates invasion and metastasis of HCC.

## Results

### Loss or reduction of TIPE2 expression in tumor tissues of HCC was significantly associated with metastasis

Our previous researches have showed that human TIPE2 was downregulated in tumor tissues of HCC compared with the paired adjacent non-tumorous tissues [10], suggesting it may be associated with HCC. Here we firstly analyzed the correlation of TIPE2 expression to clinical pathological features of HCC. As shown in Table 1, there was no significant correlation of TIPE2 expression to age, gender, cirrhosis, hepatitis B, serum AFP and pathological grade (p > 0.05). However, the TIPE2 had a significant relation to TNM stage. Rate of TIPE2 loss or low expression was higher in tumors from patients with TNM stage III-IV than that with TNM stage I-II (p = 0.044). Since TNM stage III-IV means that tumor is accompanied with vascular invasion or distant metastasis, the data suggests that the loss or low expression of TIPE2 may be associated with metastasis of HCC.

**Table 1:** Relationship of TIPE2 expression of HCC and clinical pathological parameters of patients

**Clinical pathological features**		**Number of cases**	**Expression of TIPE2**		**P value**
			*Low	**High**	
Gender	Male	105	44	61	0.961
	Female	7	3	4	
Age	<50	30	15	15	0.302
	≥50	82	32	50	
Pathological Grade	I-II	57	24	33	0.617
	III-IV	38	18	20	
Serum AFP (ng/ml)	<500	47	24	23	0.343
	≥500	37	15	22	
Cirrhosis	Absent	28	9	19	0.364
	Present	7	1	6	
Hepatitis B	Absent	14	5	9	0.46
	Present	21	5	16	
TNM stage	I-II	23	4	19	0.044
	III-IV	12	6	6	

*Low: final pathological score of TIPE2 expression as 0, +1.

High: final pathological score of TIPE2 expression as +2, +3.

### TIPE2 suppressed markedly migration and invasion of HCC cells in vitro

**Figure 1:**
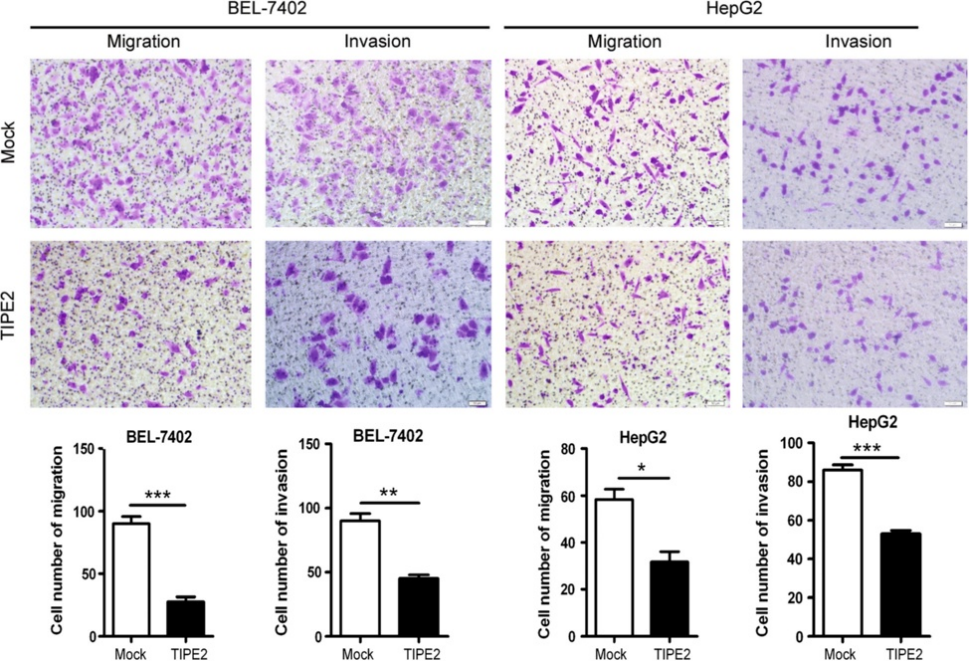
**TIPE2 decreased markedly HCC cell migration and invasion in vitro.** BEL-7402 and HepG2 cells transfected with Mock and TIPE2 plasmid were used for migration and invasion assay. Five fields of cells in the lower side were counted. Data represent means ± SEM of three independent experiments. *P < 0.05; ** P < 0.01; *** *P* < 0.001.

To explore the role of TIPE2 in HCC, we further detected the effect of TIPE2 on growth, colony formation, migration and invasion of HCC cells *in vitro*. As shown in Additional file 1: Figure S1, cells untransfected (Control) or transfected with empty plasmid (Mock) showed undetectable TIPE2 expression both on mRNA level by RT-PCR and protein level by western blot while cells transfected with TIPE2 recombinant plasmid (TIPE2) revealed high level of TIPE2 mRNA and protein. Further, overexpression of TIPE2 suppressed significantly cell viability from 48 hours and colony formation compared with Mock cell in both BEL-7402 and HepG2 (Additional file 1: Figure S1B and C). Overexpression of TIPE2 reduced markedly migration and invasion of BEL-7402 and HepG2 compared with that of Mock (Figure 1). This suppressive effect of TIPE2 was also detected in HCCLM3 with high metastasis ability (Additional file 2: Figure S2).The results indicate that TIPE2 can effectively suppress malignant phenotypes of HCC cells, especially the migration and invasion capacity *in vitro*.

### TIPE2 inhibited obviously metastasis of HCC cells in vivo

**Figure 2:**
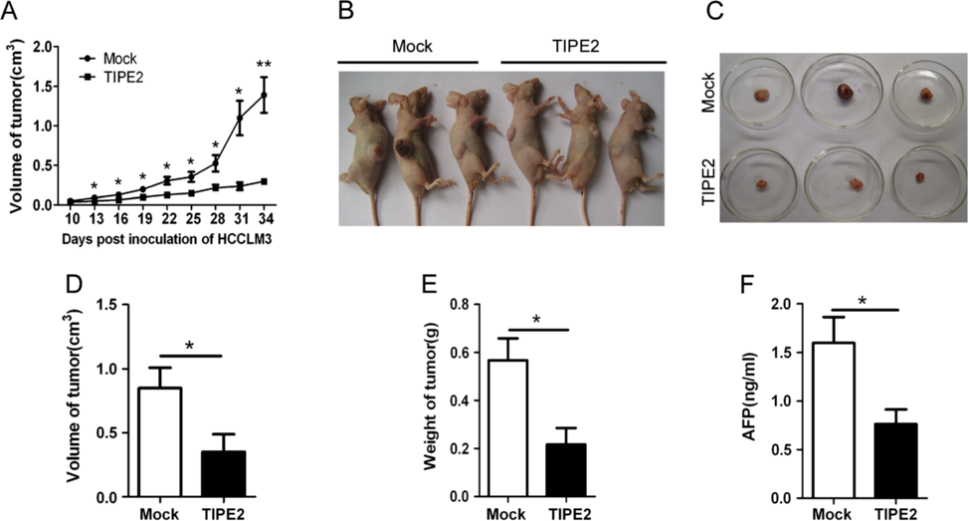
**TIPE2 suppressed effectively growth of subcutaneous xenograft tumor. (A)** The growth curves of tumors in nude mice treated by Mock and TIPE2 plasmid; **(B)** Representative image of mice with subcutaneous xenograft tumor; **(C)** Representative image of isolated tumors; **(D)** The mean tumor volume in TIPE2 group (n =5) was lower than that of Mock group (n =5); **(E)** The mean tumor weight in TIPE2 group (n =5) was lower than that of Mock group (n =5); **(F)** The mean serum AFP in TIPE2 group (n =5) was lower than that of Mock group (n =5). *P < 0.05; **P < 0.01.

**Figure 3:**
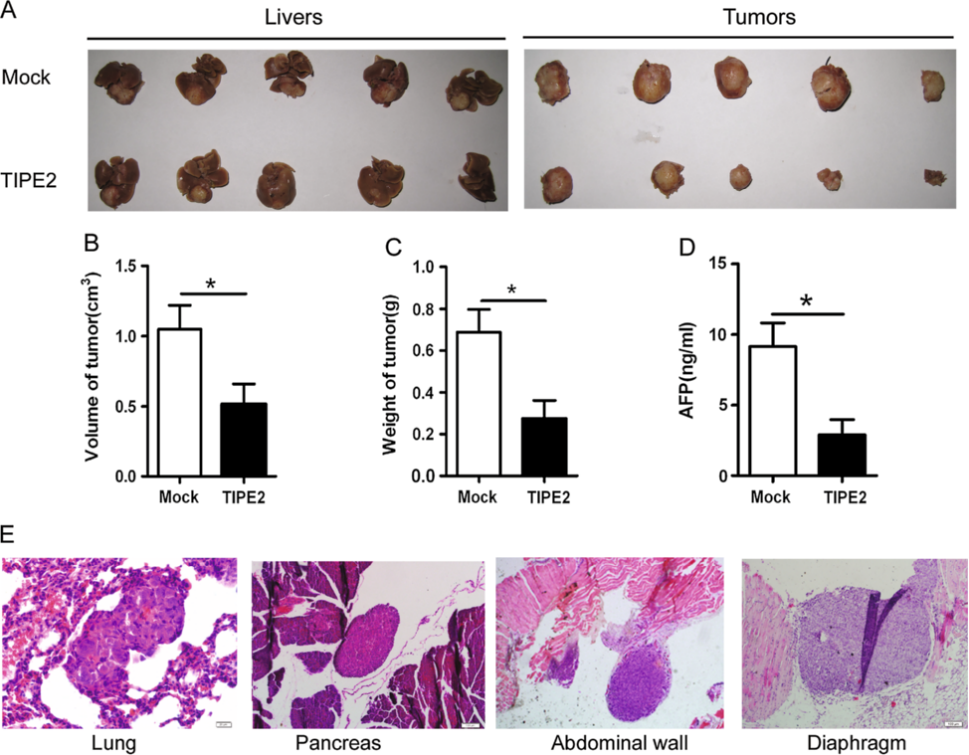
**TIPE2 inhibited growth and metastasis of xenograft in situ. (A)** Representative image of liver and tumor in mock and TIPE2 group 35 days after innoculation; **(B)** The mean tumor volume in TIPE2 group(n =5) was lower than that of Mock group (n =5); **(C)** The mean tumor weight in TIPE2 group (n =5) was lower than that of Mock group (n =5); **(D)** The mean serum AFP in TIPE2 group (n =5) was lower than that of Mock group (n =5); **(E)** Representative images of lung, pancreas, abdominal wall and diaphragm metastasis.* *P* < 0.05.

To provide *in vivo* evidence that TIPE2 suppresses HCC tumor growth and metastasis, we established two xenograft tumor models in nude mice, subcutaneous xenograft tumor model and liver orthotopic transplanted tumor model using HCCLM3 cells. In subcutaneous xenograft tumor model, we found that treatment with TIPE2 plasmid attenuated remarkably the subcutaneous tumor growth (Figure 2A). The overall mean tumor volume of TIPE2 group was much smaller than that of Mock group (Figure 2B-D). Consistent with the results, the mean tumor weight and concentration of serum AFP in TIPE2 group were also significantly lower than those of Mock group (Figure 2E and F). Moreover, the results from IHC and H&E staining showed that tumor in TIPE2 treatment group had obvious TIPE2 expression and revealed higher differentiation compared with those in the Mock group (Additional file 3: Figure S3). These results indicate that re-expression of TIPE2 in the HCC cells attenuates the tumor growth *in vivo*. Furthermore, we analyzed the effect of TIPE2 on the metastasis of HCC in the liver orthotopic implantation tumor model. As shown in Figure 3, 35 days after inoculation of xenograft tumor, tumor volume, weight and serum AFP in TIPE2 group obviously reduced compared with those in Mock group (Figure 3A-D). More importantly, 100% (5/5) of Mock group mice were detected spontaneous lung metastasis while only 60% (3/5) of TIPE2 group mice did. In addition, tumors had invaded into the pancreas, diaphragm and abdominal wall in Mock group but not in TIPE2 group (Figure 3E and Table 2). These results demonstrate that TIPE2 markedly suppresses growth and metastasis of HCC cells *in vivo*.

**Table 2:** Metastasis rate of orthotopic transplanted HCC in different organs or tissure

**Organs or tissue**	**Metastasis rate**	
	**Mock**	**TIPE2**
Lung	100%(5/5)	60%(3/5)
pancreas	40%(2/5)	0(0/5)
Diaphragm	20%(1/5)	0(0/5)
Abdominal wall	20%(1/5)	0(0/5)

### TIPE2 suppressed migration and invasion of HCC cell via inhibiting Rac1 pathway

**Figure 4:**
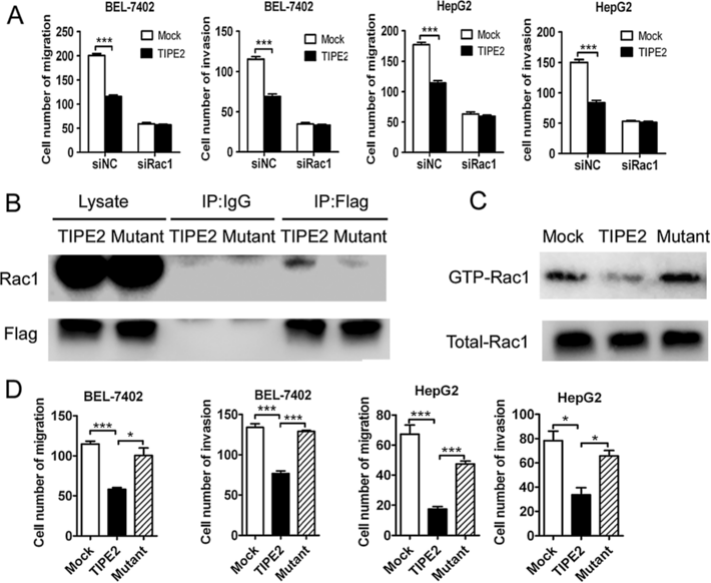
**TIPE2 suppressed HCC migration and invasion via inhibiting Rac1 pathway. (A)** BEL-7402 and HepG2 cells that were cotransfected with Mock or TIPE2 plasmids and siNC or siRac1 were used for migration and invasion assay. **(B)** BEL-7402 cell was transfected with TIPE2 or mutant plasmids for 24 h, the cell lysates were prepared and immonoprecipitated with anti-Flag antibody or isotype IgG. The precipitates and cell lysates were subjected to Western blotting with anti-Flag and anti-Rac1 antibody; **(C)** BEL-7402 cell were transfected with Mock, wide type TIPE2 and mutant TIPE2 plasmids respectively, and cell lysates were subjected to pull down using PAK-GST protein beads. Activated Rac1 from pull down and total Rac1 in the lysates were detected by Western blot; **(D)** BEL-7402 and HepG2 cells that were transfected with Mock, TIPE2 and Mutant TIPE2 plasmid were used for migration and invasion assay. Data shown are representative of three independent experiments (n = 3). *P < 0.05; *** *P* <0.001.

The results above suggest that TIPE2 is able to inhibit metastasis and invasion of HCC *in vitro* and *in vivo*. Next, we want to know how TIPE2 impact them. Our previous report has demonstrated that murine TIPE2 is able to inhibit F-actin polymerization and cell migration by targeting RGL, one of Ras signaling pathway effectors [10]. However, we found that expression of RGL was very low in all HCC cell lines detected (Additional file 4: Figure S4A), suggesting RGL may be not a major target of TIPE2 in HCC. More recent research showed that TIPE2 regulated negatively murine innate immunity by binding and blocking Rac1GTPases [8]. So we hypothesized that TIPE2 may suppress the migration and invasion via targeting Rac1 in HCC. To test this hypothesis, we undertook approaches as follows. First, we detected expression of Rac1 and its action in migration and invasion of HCC. Consistent with previous researches, high level of Rac1 expression were detected in multiple cell lines and its expression was positively related with metastasis capacity (Additional file 4: Figure S4A). To determine whether TIPE2 regulates migration and invasion through Rac1, we manipulated the Rac1 activity using chemical blocker NSC23766. The Rac1 antagonist NSC23766 attenuated cell migration and invasion markedly, and effectively reduced the difference between mock and TIPE2 group at 50 μM both in BEL-7402 and HepG2 (Additional file 4: Figure S4C). Consistent with this, silence of Rac1 expression by specific siRNA abolished the inhibition effect of TIPE2 on migration and invasion of tumor cells (Figure 4A). Furthermore, coimmunoprecipitation (co-IP) revealed that human wild type TIPE2 could bind to Rac1 while mutant TIPE2 lost the ability in HCC cell (Figure 4B). Additionally endogenous human TIPE2 was also able to bind to Rac1 in human monocyte cell line Thp-1 (Additional file 4: Figure S4B). Using a GST pull-down assay, we found that transfection of wild type TIPE2 decreased the Rac1GTPase activity while mutation of TIPE2 reversed its inhibition effect, indicating that human TIPE2 can also bind and block Rac1 activity in HCC (Figure 4C). Third, mutation of TIPE2 reversed inhibiting effect of wild type TIPE2 on migration and invasion both in BEL-7402 and HepG2 (Figure 4D). All the results indicate that TIPE2 suppresses migration and invasion of HCC cells via inhibiting Rac1 pathway.

### TIPE2 suppressed F-actin polymerization and MMP9, uPA expression via inhibiting Rac1 pathway

**Figure 5:**
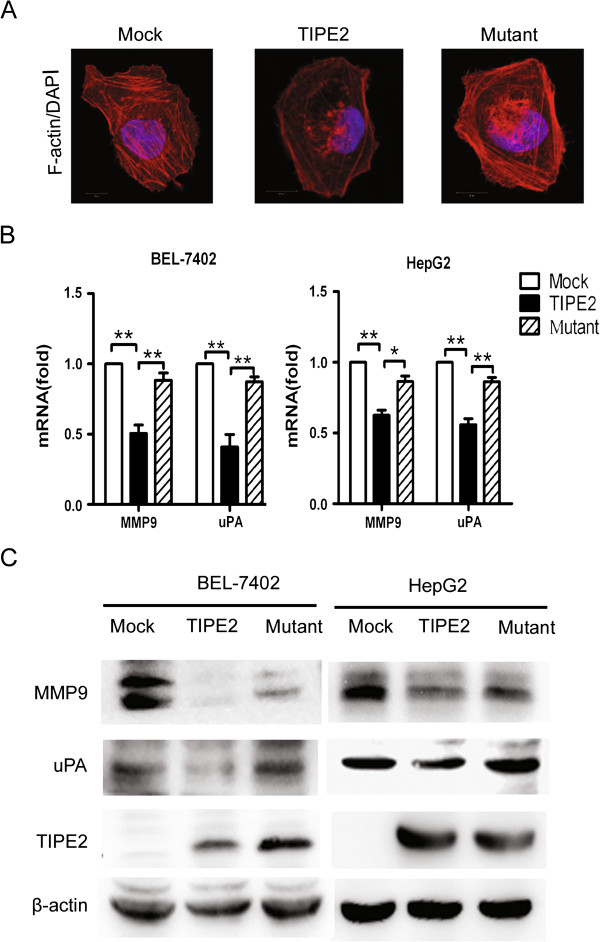
**TIPE2 decreased F-actin polymerization and expression of MMP9 and uPA via inhibiting Rac1 pathway. (A)** After transfected with mock, TIPE2 and Mutant TIPE2 plasmids respectively, the microfilament in 7402 cell was stained with Tetramethylrhodamine (TRITC)-conjugated phalloidin and observed under laser confocal microscopy; **(B)** MMP9 and uPA levels in BEL-7402 and HepG2 were detected by Real time PCR; **(C)** The protein levels of MMP9 and uPA in BEL-7402 and HepG2 were detected by western blot. Data shown are representative of three independent experiments (n = 3). *P < 0.05; **P < 0.01.

It has been known that microfilaments play a key role in cytoskeleton reorganization and in cell motility and tumor metastasis potential [13]. Therefore, we examined the organization of microfilaments in cells transfected with Mock, wild type TIPE2 or mutant TIPE2 plasmid by immunofluorescence method. Compared with Mock group, there was an obvious microfilament depolymerization in TIPE2 group (Figure 5A) but this action was reversed by TIPE2 mutation. In addition, the MMP9 and uPA are responsible for the degradation of extracellular matrix components and play important roles in the process of cancer invasion and metastasis [14,15]. Therefore, we further examined the effect of TIPE2 on expression of MMP9 and uPA expression. As shown in Figure 5B and C, wild type TIPE2 obviously attenuated MMP9 and uPA expression on mRNA and protein level but TIPE2 mutation lost this ability. Additionally, we found that expression of MMP9 and uPA also decreased after silence of Rac1 (Additional file 4: Figure S4 D, E). All the results indicate that TIPE2 decreases F-actin polymerization and expression of MMP9 and uPA via inhibiting Rac1 pathway.

## Discussion

Although murine TIPE2 has been partly characterized, the information about human TIPE2 is limited. In present study, we provide novel evidences for first time that human TIPE2 is able to suppress effectively the migration and invasion of HCC *in vitro* and *in vivo* and further indicate that human TIPE2 is endogenous inhibitor of Rac1 in HCC by which it reduces F-actin polymerization and expression of MMP9 and uPA.

The TIPE (TNFAIP8) family is a recently identified group of proteins including four members, TNFAIP8, TIPE1 (TNFAIP8L1), TIPE2 (TNFAIP8L2), and TIPE3 (TNFAIP8L3) [3]. Increasing experimental evidences support that TNFAIP8 is an oncogene in human cancers, such as breast cancer and lung cancer [16,17]. TIPE1 is supposed that it may play role in carcinogenesis for a high level of TIPE1 mRNA is detected in most human carcinoma cell lines [18]. However, here we demonstrate that human TIPE2 plays an inhibitory role in HCC. We find that : (1) The forced expression of TIPE2 in HCC-derived cell lines (such as BEL-7402, HepG2) markedly inhibits tumor growth, colony formation, migration and invasion in vitro; (2) More importantly, in subcutaneous xenograft tumor and liver orthotopic transplanted nude murine models, the restoration of TIPE2 expression in HCC cells (HCCLM3) with high metastasis potential markedly suppresses the progress of HCC, invasion to adjacent organ and spontaneous lung metastasis; (3) The loss or reduced expression of TIPE2 expression in primary HCC tissues is significantly associated with tumor metastasis. Taken together, we indicate that TIPE2, unlike other members of TIPE family as oncogene in various cancers, is a tumor suppressor, at least in HCC. The results suggest that despite structural relation of TIPE family, members of the family possess diverse functions. In addition, in contrast to murine TIPE2 which is easy to be lost in tumor, expression of human TIPE2 is relatively stable in tumor. Our previous researches showed that although stably expression of murine TIPE2 in Ras 3 T3 cell line significantly delayed tumor onset, TIPE2 tumors, once formed, could grow to the same weight as control because of ubiquitin-mediated degradation of TIPE2 protein in tumor cells [10]. In present research, after last TIPE2 plasmid injection, high level of TIPE2 expression was detected in subcutaneous tumor (Additional file 3: Figure S3A). And after the tumor was transplanted into liver for 35 days, TIPE2 expression was still slightly detectable in some of tumor tissues (Additional file 5: Figure S5). These results suggest that forced expression of human TIPE2 provides a possibility for treatment of HCC in future.

Rac1, which has been shown to be involved in cancer cell metastasis, is highly expressed in aggressive HCC cell lines and its activity correlated with cell motility and cytoskeleton polymerization [13,19]. Furthermore, Rac1 regulates various downstream effective molecules related with metastasis, such as MMP9 [20] and uPA [21]. Rac1 can be activated by a variety of stimuli, including growth factors (such as EGF and PDGF) [22,23] and virus infection (HBV) [24]. Researches about endogenously negative regulation of Rac1 are advancing. Overexpression of miR-142-3p decreases Rac1 mRNA and protein levels, implying miR-142-3p negatively regulates Rac1 via inhibiting its translation [25]. Plexin-B1 binds to active Rac1 and functions as a negative regulator of the RacGTPases in mouse macrophages [26]. Here, we found that overexpression of TIPE2 markedly attenuated the activity of Rac1. The mutation of TIPE2 in α0 domain reduced its ability to bind Rac1 and subsequently inhibited Rac1 activity. Coordinately, the restoration of Rac1 activity after TIPE2 mutation reversed the suppression of wild type TIPE2 to HCC cell migration and invasion. Meanwhile, downstream effective molecules of Rac1, F-actin polymerization and expression of MMP9 and uPA are reversed by TIPE2 mutation. These results indicate that human TIPE2 is endogenous inhibitor of Rac1 in liver cells by which it controls the activity of Rac1 under physiological condition. This is also supported by the direct binding of murine TIPE2 to Rac1 and inhibition of its activity in innate immune cell [8].

## Conclusion

Collectively, human TIPE2 is an endogenous inhibitor of Rac1 by which it functions as a tumor suppressor to control cell proliferation, migration and invasion. Loss or decreased TIPE2 expression results in upregulation of Rac1 activity which promotes the migration and invasion of HCC. The forced expression of human TIPE2 is a new strategy for treatment of HCC.

## Methods

### Patients

One hundred and twelve primary hepatocellular carcinoma specimens were used for detection of TIPE2 expression. Of them, 77 specimens were from HCC tissue chip (OUTDO BIOTECH, Shanghai, China). Other 35 specimens were obtained from patients who underwent operations at Qilu Hospital of Shandong University. These human procedures were preapproved by the Institutional Review Board of the Shandong University and were approved by the Institutional Review Board of the Shandong University.

### Cell culture

The human HCC cell lines, BEL-7402, HepG2, SMMC-7721 and human monocyte cell line Thp-1 were purchased from Shanghai Cell Bank of Chinese Academy of Sciences (Shanghai, China), grown in RPMI 1640 medium (Gibco, CA, USA) supplemented with 10% heat-inactivated fetal bovine serum (FBS) (Gibco). HCCLM3 cell line was purchased from Liver Cancer Institute, Zhongshan Hospital (Shanghai, China), grown in DMEM medium (Gibco) supplemented with 10% FBS.

### Plasmid construction, siRNA and transfection

Full-length human TIPE2 was generated from the cDNA clone by PCR and cloned in frame with a C-terminal Flag into vector PRK5.The mutant TIPE2 in which the TIPE2 N-terminal lysine or arginine residues, Lys-15, Lys-16, and Arg-24 were replaced with glutamine or alanine was generated by PCR-based site-directed mutagenesis as previously described [8]. Specific siRNA for Rac1 and nonspecific negative control were purchased from Sigma-Aldrich (Louis, USA). Transfection of tumor cells with plasmid or siRNA was performed using Lipofectamine 2000 according to the manufacturer’s protocols (Invitrogen, Carlsbad, CA, USA).

### RNA isolation, RT-PCR and real-time PCR

Total RNA was extracted from cells using Trizol Reagent (Invitrogen). Semi-quantative RT-PCR was performed using 2 × Taq MasterMix (CWBIO, Beijing, China). Real-time PCR was performed using UltraSYBR Mixture (CWBIO). The sequences of the sense and antisense primers were as follows: MMP9: 5′-GCATTCAGGGAGACGCCCATTT AACGACA-3′, and 5′-CTGACACTCCCGGTGGG AAATCA-3′; TIPE2: 5′-ACTGA GTAAGATGGCGGGTCG-3′, and 5′-TTCTGGCGAA AGCGGGTAG-3′; Rac1: 5′-AT GTCCGTGCAAAGTGGTATC-3′, and 5′-CTCGGATCGCTTCGTCAAACA-3′; GAPDH: 5′-AACGGATTTGGTCGTATTGGG-3′, and 5′-CCTGGA AGATGGTGAT GGGAT-3′. Relative levels of gene expression were determined with GAPDH as the control.

### Western blot

Equal amount of protein was separated by SDS-PAGE and transferred onto PVDF membranes (Millipore, Billerica, MA, USA). Membranes were probed overnight at 4°C with the following primary antibodies: rabbit polyclonal antibody against human TIPE2 (1:300; BOSTER, Wuhan, China), rabbit monoclonal antibody against the matrix metalloproteinase 9 (MMP-9) and urokinase-PA (u-PA) (1:1000; EPITOMICS, Hangzhou, China), mouse monoclonal antibody against RGL1 (1:200; Santa Cruz, CA, USA), Rac1 (1:300; abcam, Hongkong), β-actin (1:1000; ZSGB-Bio, Beijing, China), followed by secondary antibodies (1:2000; goat anti rabbit or mouse IgG, ZSGB-Bio) conjugated with peroxidase for 1 h at room temperature. After washing, signals were visualized by eECL Western Blot Kit (CWBIO).

### Immunohistochemistry (IHC)

The paraffin slides were stained with rabbit antibody against TIPE2 (1:200) at 4°C overnight. Secondary staining was performed with HRP-conjugated anti-rabbit IgG using a MaxVsion Kit and 3, 5-diaminobenzidine (DAB) peroxidase Substrate Kit (Maixin Co., Fuzhou, China). The sections were counterstained with hematoxylin. Isotope-matched human IgG was used as a negative control. All IHC staining was independently assessed by two experienced pathologists. The staining intensity was scored from 0 to 3 (0, no staining; 1, weak; 2, moderate; 3, strong). The staining extent was scored from 0 to 3 based on the percentage of positive cells (0, < 1%; 1, 1%-33%; 2, 34%-66%; 3, 67%-100%). The two scores for each slide were then combined to produce a final grade of TIPE2 expression: 0, total score = 0; 1+, total score = 1–2; 2+, total score = 3–4; 3+, total score = 5–6. When there were discrepancies between the two pathologists, the average score was used.

### Colony formation assay

Cells were seeded in six-well plates at a density of 1500 cells per well for 1–2 weeks and then fixed with 20% methanol and stained with 1% crystal violet. Colonies that is consisted of more than 50 cells were counted and calculated as a percentage of that of the control group. The experiment was independently performed for three times.

### Cell viability assay

Cells were seeded in 96-well plates at 5000 cells/well and cultured for indicated time points. Cell viability was evaluated using CCK8 (Beyotime, Haimen, China) according to manufacturer’s instructions. The absorbance was determined at 450 nm wave length. Each time point was repeated in three wells and the experiment was independently performed for three times.

### Transwell assay for cell migration and invasion

Tumor cell migration and invasion were analyzed in 24-well Boyden chambers with 8-μm pore size polycarbonate membranes (Costar, Acton, USA). For invasion assay, the membranes were precoated with 50 μg Matrigel (BD Biosciences, San Diego, USA) to form matrix barriers. Cells (1 × 10^5^) were resuspended in 100 μl serum-free medium and placed in the upper chamber, and the lower compartments were filled with 600 μl medium with 10% FBS. After incubation, the cells remaining on the upper surface of the membrane were removed. The cells on the lower surface of the membrane were fixed and stained with crystal violet and counted under a light microscope at ×200 magnification. In some experiments, Rac1 inhibitor, NSC23766 (Calbiochem, San Diego, USA) was used to inhibit Rac1 activity.

### Immunofluorescence (IF)

The cells on the cover slip were fixed, permeabilized and then stained with Tetramethylrhodamine (TRITC)-conjugated phalloidin (Sigma–Aldrich, Louis, USA) for 1 h. Nuclei were stained by 4′,6-diamidino-2-phenylindole (DAPI) (Beyotime) for 5 min. Results were analyzed on a confocal laser microscopy (Carl Zeiss, LSM780, Oberkochen, Germany).

### Establishment of orthotopic transplanted nude mice model of human HCC matastasis

Male athymic BALB/c nu/nu mice (4–6 week old) were purchased from Chinese Academy of Sciences (Shanghai, China) and maintained in laminar-flow cabinets under specific pathogen-free conditions. For evaluation of the tumor growth *in vivo*, 1 × 10^7^ HCCLM3 cells in 100 μl of PBS were injected subcutaneously into flank of nude mice (n = 10). When tumors reached approximately 1 mm^3^ in size 10 day later, these mice were randomly divided into two groups, Mock group (n = 5) was treated with 20 μg of empty plasmid and TIPE2 group (n = 5) with TIPE2 plasmid by intra-tumor injection every 3 days, respectively. The tumor size was measured and the tumor volume was calculated as follows: length × width × width × 0.4. After another 25 days, the implanted tumors were dissected and cut into pieces of around 1 × 1 × 1 mm^3^ and transplanted into liver parcel of other nude mice (n = 5 for each group). The mice were sacrificed 35 days after innoculation. All possible metastasizing visceral organs or tissures including lungs, pancreas, diaphragm and abdominal wall were removed and processed for standard histopathological study. Serial sections were made for every tissure. Any slide with metastasis was assumed as positive. The serum AFP was detected on electro-chemiluminescence immunoassay systerm (Roche, Cobas E601, Germany).

### Co-Immunoprecipitation (co-IP)

Cell lysate was prepared as previously described [8]. One μl mouse monoclonal antibody against Flag (HuaAn, Hangzhou, China), Rac1 or isotype IgG was added to 1 ml of cell lysate and incubated for 1 h at 4°C and 20μl of resuspended Protein A/G Plus-Agrose (Santa Cruz) was added to the above mixture and incubated for 4 h at 4°C with rotation. The pellet was washed four times with PBS and boiled in 2× Laemmli buffer. The proteins were detected by western blot.

### PBD pull-down assay

Cell lysate was prepared as previously described [8]. The lysate was incubated with 20 μg of p21-activated kinase (PAK)-GST protein beads (Cytoskeleton) for 30 min at 4°C. After washing, proteins on beads and in total cell lysates were subjected to western blot to determine the level of active Rac1.

### Statistical analysis

Statistical analysis was performed with SPSS 13.0 (SPSS Inc., Chicago, USA). The wilcoxon test was employed to compare qualitative variables while the Student *t* test for quantitative variables. All statistical tests were two-sided and *P* value < 0.05 was considered statistically significant for all tests.

## Abbreviations

HCC: Hepatocellular carcinoma; TIPE2: Tumor necrosis factor (TNF)-alpha-induced protein 8-like 2; IHC: Immunohistochemistry; RT-PCR: Reverse-transcriptase polymerase chain reaction; co-IP: Co-immunoprecipitation; MMP: Matrix metallopeptidase; uPA: Urokinase plasminogen activator; AFP: Alpha-fetoprotein.

## Competing interests

The authors declare that they have no competing interests.

## Authors’ contribution

LZ designed research and analyzed data. XC, LZ, YS, YS, SD performed experiments. CG, FZ, QW performed statistical analysis. JW and XW carried out the pathologic analysis. YHC made contributions to the conception and design of experiments. XC and ZL wrote the manuscript. All authors read and approved the final manuscript.

## Supplementary Material

Additional file 1: Figure S1 TIPE2 suppressed HCC cell proliferation and colony formation. (A) TIPE2 mRNA and protein expression in two HCC line (BEL-7402, HepG2) were examined by RT-PCR and western blot after transient transfection with Mock and TIPE2 plasmid; (B) After transfection with TIPE2 or Mock plasmid, BEL-7402 and HepG2 were reseeded in 96-well plate. Cell viability was assessed by CCK8 method at indicated time points. (C) Colonies that consisted of more than 50 cells were counted and calculated as a percentage of that of the control group. The experiments were independently performed for three times. *P < 0.05; ** *P* < 0.01. Click here for file

Additional file 2: Figure S2 TIPE2 decreased markedly HCCLM3 cell migration and invasion in vitro. HCCLM3 transfected with mock or TIPE2 plasmid were used for migration and invasion assay. Data shown are representative of three independent experiments. *** *P* <0.001. Click here for file

Additional file 3: Figure S3 Histopathology of *subcutaneous xenograft tumors*. (A) Expressions of TIPE2 in tumor tissue was detected by immunohistochemistry; (B) Representative image of tumor tissue in Mock and TIPE2 group by H&E staining. Click here for file

Additional file 4: Figure S4 Rac1 was correlated with the migration and invasion of HCC cell. (A) The expression of Rac1 as well as RGL in different cell lines (BEL-7402, HepG2, SMMC-7721, HCCLM3) were detected by western blot; (B) The cell lysates of Thp-1 was prepared and immonoprecipitated with anti-Rac1 antibody or isotype IgG. The precipitates and cell lysates were subjected to Western blotting with anti-TIPE2 and anti-Rac1 antibody respectively; (C) BEL-7402 and HepG2 cells pretreated with Rac1 inhibitor NSC23766 (50 μM) and transfected with Mock or TIPE2 plasmids were used for migration and invasion assay; (D) MMP9, uPA and Rac1 levels were detected by real time PCR. (E) MMP9, uPA and Rac1 levels were detected by western blot. Data shown are representative of three independent experiments. **, P < 0.01; *** P < 0.001. Click here for file

Additional file 5: Figure S5 TIPE2 was slightly detectable in *liver orthotopic* tumor tissue. Expression of TIPE2 in tumors was detected by immunohistochemistry. Representative images were shown. Click here for file
