# Supplementary figures and images for: Human tumor necrosis factor (TNF)-alpha-induced protein 8-like 2 suppresses hepatocellular carcinoma metastasis through inhibiting Rac1
Source: Mol Cancer. 2013 Nov 26;12:149. doi: 10.1186/1476-4598-12-149 (PMC4176125; doi:10.1186/1476-4598-12-149)

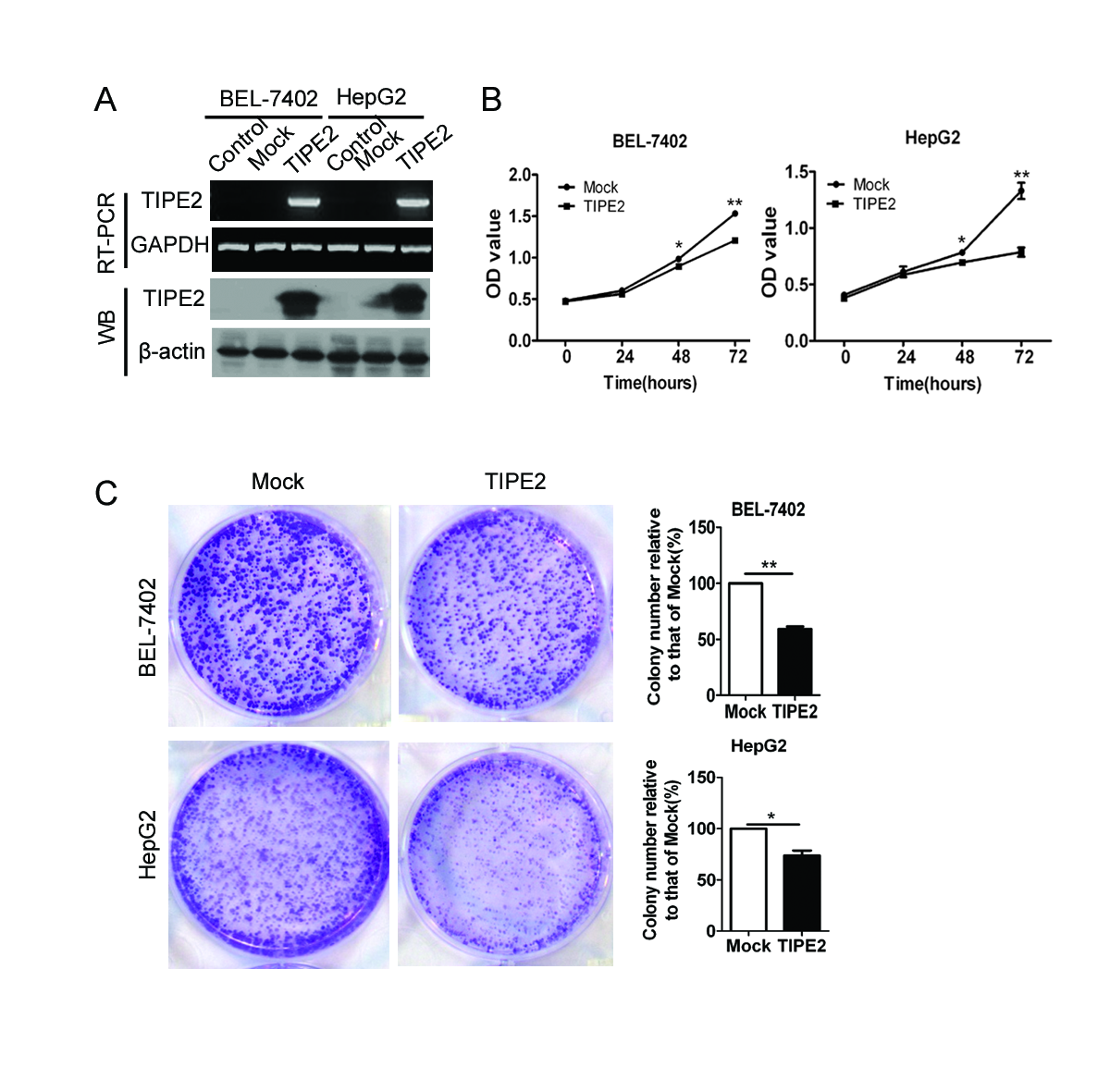

Supplement: Additional file 1: Figure S1 — TIPE2 suppressed HCC cell proliferation and colony formation. (A) TIPE2 mRNA and protein expression in two HCC line (BEL-7402, HepG2) were examined by RT-PCR and western blot after transient transfection with Mock and TIPE2 plasmid; (B) After transfection with TIPE2 or Mock plasmid, BEL-7402 and HepG2 were reseeded in 96-well plate. Cell viability was assessed by CCK8 method at indicated time points. (C) Colonies that consisted of more than 50 cells were counted and calculated as a percentage of that of the control group. The experiments were independently performed for three times. *P < 0.05; ** P < 0.01. [file 1476-4598-12-149-S1.tiff]

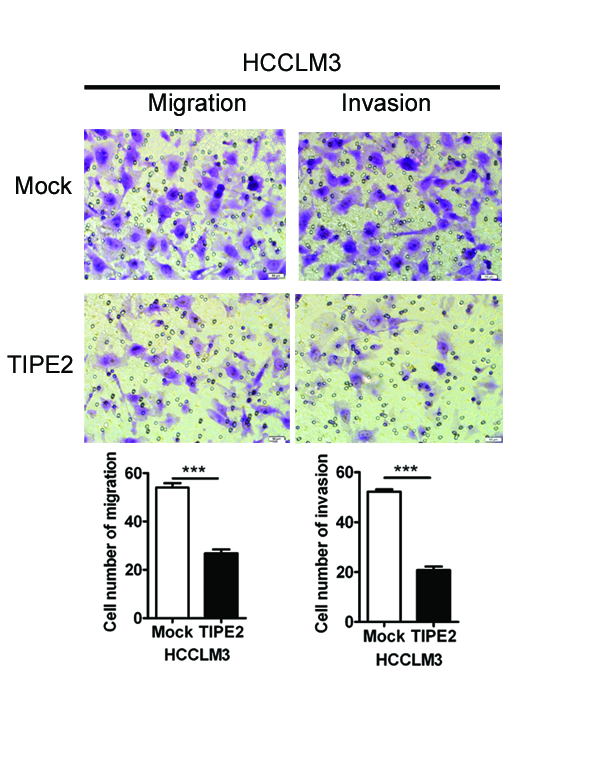

Supplement: Additional file 2: Figure S2 — TIPE2 decreased markedly HCCLM3 cell migration and invasion in vitro. HCCLM3 transfected with mock or TIPE2 plasmid were used for migration and invasion assay. Data shown are representative of three independent experiments. *** P <0.001. [file 1476-4598-12-149-S2.tiff]

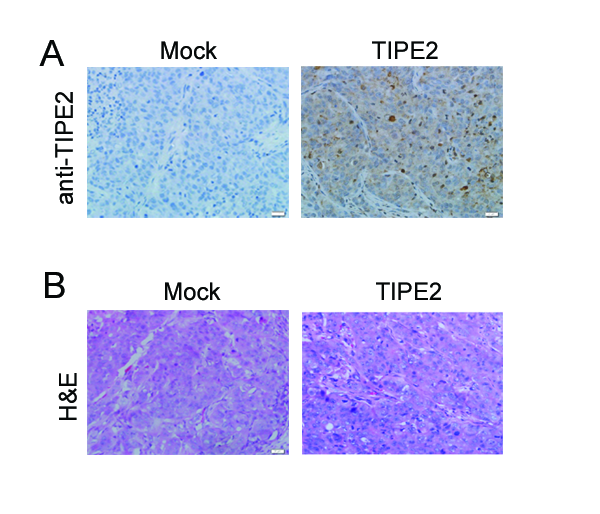

Supplement: Additional file 3: Figure S3 — Histopathology of subcutaneous xenograft tumors. (A) Expressions of TIPE2 in tumor tissue was detected by immunohistochemistry; (B) Representative image of tumor tissue in Mock and TIPE2 group by H&E staining. [file 1476-4598-12-149-S3.tiff]

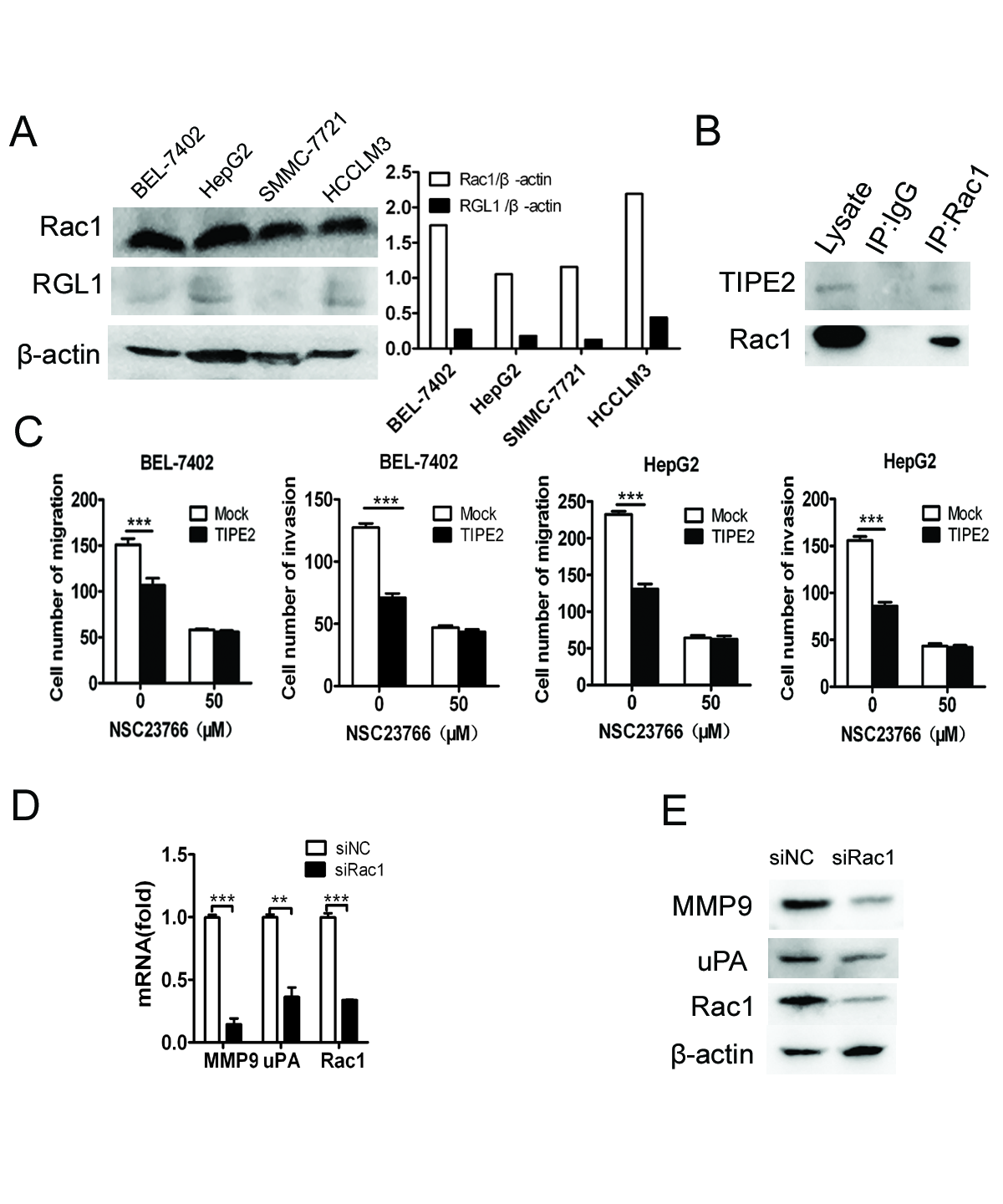

Supplement: Additional file 4: Figure S4 — Rac1 was correlated with the migration and invasion of HCC cell. (A) The expression of Rac1 as well as RGL in different cell lines (BEL-7402, HepG2, SMMC-7721, HCCLM3) were detected by western blot; (B) The cell lysates of Thp-1 was prepared and immonoprecipitated with anti-Rac1 antibody or isotype IgG. The precipitates and cell lysates were subjected to Western blotting with anti-TIPE2 and anti-Rac1 antibody respectively; (C) BEL-7402 and HepG2 cells pretreated with Rac1 inhibitor NSC23766 (50 μM) and transfected with Mock or TIPE2 plasmids were used for migration and invasion assay; (D) MMP9, uPA and Rac1 levels were detected by real time PCR. (E) MMP9, uPA and Rac1 levels were detected by western blot. Data shown are representative of three independent experiments. **, P < 0.01; *** P < 0.001. [file 1476-4598-12-149-S4.tiff]

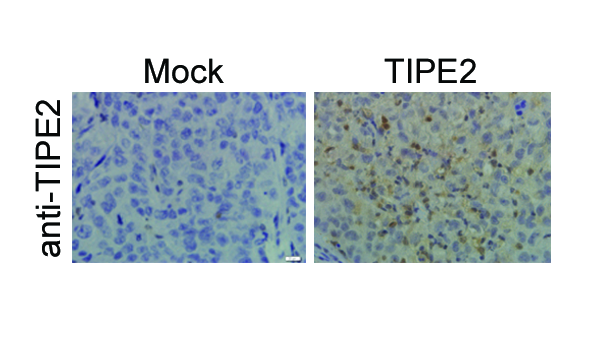

Supplement: Additional file 5: Figure S5 — TIPE2 was slightly detectable in liver orthotopic tumor tissue. Expression of TIPE2 in tumors was detected by immunohistochemistry. Representative images were shown. [file 1476-4598-12-149-S5.tiff]
